# Peer Review of “Predicting Waist Circumference From a Single Computed Tomography Image Using a Mobile App (Measure It): Development and Evaluation Study”

**DOI:** 10.2196/54012

**Published:** 2023-12-12

**Authors:** Mohammed Shahriar Arefin

**Affiliations:** 1 Department of Bioengineering, Temple University, Philadelphia, PA, United States

**Keywords:** waist circumference, computed tomography, abdominal CT, mobile health, health apps, CT, CT scan, CT image, mobile app, app, application, waist, body, body mass, BMI, morbidity, mortality, clinical, tool, prototype, design, obesity, abdominal, usability, validity, medical

*This is a peer-review report submitted for the paper “Predicting Waist Circumference From a Single Computed Tomography Image Using a Mobile App (Measure It): Development and Evaluation Study.”*

## Round 1 Review

### General Comments

The authors created a mobile app that predicts waist circumference (WC) from computed tomography (CT) images [1]. After creating the app, the authors conducted a preliminary study involving 20 patients. The results showed that the developed app can predict WC from CT images with high accuracy. Though the paper showed some promising results, the authors still need to clarify a few important points. I hope the authors would be happy to clarify those points.

### Specific Comments

#### Major Comments

 1. What was the primary reason for selecting equation 1 as a reference method for WC calculation? Isn’t it possible to calculate the exact circumference from the CT images using image-processing algorithms? Wouldn’t it be more representative compared to the manual WC detection procedure?
 2. Keeping the mobile app aside, how much different is this study compared to Ciudin et al [2]?
 3. On page 3, please expand the discussion on “App Requirements.” It is not evident what was meant by “app requirements” in this section.
 4. How many images were taken from each CT slice? As the measurements for the waist parameters (a and b) were taken using a manual process, what kind of procedure was followed to ensure that the person-to-person variability remains low?
 5. In Figures 3 and 4, there is a small dot around the top of the figures. Is this a data point? Additionally, proper x- and y-axis labels are missing. Please add appropriate units on the x- and y-axis.
 6. In the Discussion section, it was claimed that “this is the first of a kind mobile app helping physicians to estimate WC.” Do the authors think the physicians would be able to use apps such as [3] to assess WC?
 7. In the Discussion section, it was stated that “Moreover, the simplicity of the app may reduce the time required for physicians to assess WC.” How fast is the app compared to the manual approach?

#### Minor Comments

 8. The authors stated that “WC cannot be physically assessed in patients with intellectual or motor disabilities” but did not provide any other details as to why it can’t be assessed. The authors should discuss this in detail in the Introduction.
 9. The sentence “However, for a radiologist, this method requires training and can be more or less time consuming” seems confusing. If possible, please restructure this sentence.
 10. In equation 1, what is denoted by “p”?
 11. Although the authors discussed in the Methods section how the measurements were taken just above the iliac crest and the CT images were taken from the last slice to ensure that those are not taken from different places, do the authors think that there could be some positional errors being introduced based on your approach?
 12. On page 3, it was stated that there were further modifications to the app design. What kind of modifications were carried out? Did the authors discard the prior mobile app–based WC measurements (mWCs) after modifying the app?
 13. Please try to make sure that periods and commas are being used appropriately. On page 4, one of the sentences was “The mean BMI was 26±4; 27,8±2,7 for women and 24.2±4,4 for men.” For women, a comma was used as a decimal point. On the other hand, for men, a period was used as a decimal point.
 14. In Table 1, what is the unit for “Confidence Interval”?
 15. What kind of procedure was used to perform the diagnostic test to detect abdominal obesity? Please discuss this in the Methods section.

## Round 2 Review

Thanks to the authors for providing a detailed revised version and comments.

If the authors can clear up a few more confusions, then it would be great.

### Minor Comments

 1. The authors stated that the app has an accuracy of 83% when using the mWC to detect abdominal obesity. Is it sufficient compared to the conventional approaches? Just a simple comparison/comment would suffice.
 2. Related to comment 11 of the round 1 review, how much impact can positional errors have in abdominal obesity classification? This can be explained or discussed in the Discussion.
 3. The Figure 3 regression shows that one of the app measurements was (WC_App=120) when the true value should have been around ~65 (standing app difference=55). But in Figure 4, that point seems to be missing (mean of standing + app ~92, so the difference ~55 should be around ~92 in the Bland-Altman plot). Can you please clarify this? If my calculations are wrong, I am extremely sorry about that.

Overall, the authors discussed all of the comments raised by the reviewer.

## Abbreviations

CT: computed tomography
mWC: mobile app–based waist circumference measurement
WC: waist circumference
